# Overwintering evergreen oaks reverse typical relationships between leaf traits in a species spectrum

**DOI:** 10.1098/rsos.160276

**Published:** 2016-07-20

**Authors:** Hisanori Harayama, Atsushi Ishida, Jin Yoshimura

**Affiliations:** 1 Hokkaido Research Center, Forestry and Forest Products Research Institute, Hitsuji-gaoka, Toyohira-Ku, Sapporo, Hokkaido 062-8516, Japan; 2 Center for Ecological Research, Kyoto University, Hirano, Otsu, Shiga 520-2113, Japan; 3 Graduate School of Science and Technology and Department of Mathematical and Systems Engineering, Shizuoka University, Johoku, Naka-Ku, Hamamatsu, Shizuoka 432-8561, Japan; 4 Department of Environmental and Forest Biology, State University of New York College of Environmental Science and Forestry, Syracuse, NY 13210, USA; 5 Marine Biosystems Research Center, Chiba University, Kamogawa, Chiba 299-5502, Japan

**Keywords:** leaf economics spectrum, leaf nitrogen, photosynthesis, *Quercus*, winter cold

## Abstract

The leaf economics spectrum has given us a fundamental understanding of the species variations in leaf variables. Across plant species, tight correlations among leaf mass per area (LMA), mass-based nitrogen (*N*_m_) and photosynthetic rate (*A*_m_) and leaf lifespan have been well known as trade-offs in leaf carbon economy. However, the regional or biome-level correlations may not be necessary to correspond with the global-scale analysis. Here, we show that almost all leaf variables in overwintering evergreen oaks in Japan were relatively well included within the evergreen-broadleaved trees in worldwide temperate forests, but *N*_m_ was more consistent with that in deciduous broadleaved trees. Contrary to the universal correlations, the correlation between *A*_m_ and *N*_m_ among the evergreen oaks was negative and the correlation between *A*_m_ and LMA disappeared. The unique performance was due to specific nitrogen allocation within leaves, i.e. the evergreen oaks with later leaf maturation had lower *N*_m_ but higher nitrogen allocation to photosynthetic enzymes within leaves, to enhance carbon gain against the delayed leaf maturation and the shortened photosynthetic period due to cold winters. Our data demonstrate that correlations between leaf variables in a local scale are occasionally different from averaged global-scale datasets, because of the constraints in each biome.

## 1. Introduction

**Figure 1.**
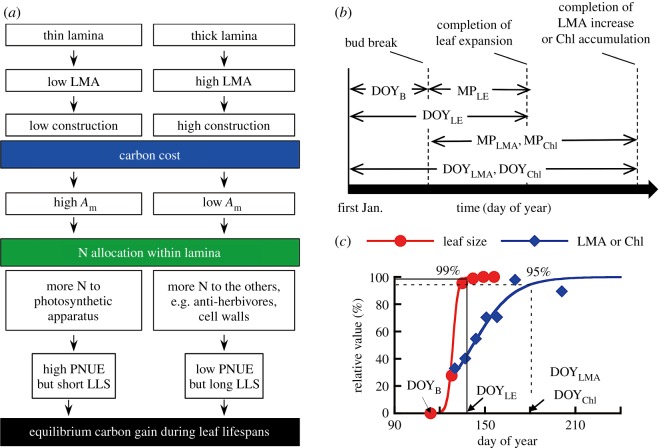
General trade-offs regarding leaf carbon economy (*a*) and schematics of the leaf maturation process in current-year leaves (*b*,*c*). Panel (*a*) shows the generally recognized trade-offs in carbon economy. LMA is the leaf mass per area, *N*_m_ is the mass-based nitrogen within the leaves, *A*_m_ is the mass-based assimilation rate, PNUE is the nitrogen-based assimilation rate and LLS is the leaf lifespan. Panel (*b*) shows the calendar dates of leaf-bud break (DOY_B_), cessation of leaf expansion (DOY_LE_), cessation of LMA increase (DOY_LMA_), cessation of chlorophyll (Chl) accumulation (DOY_Chl_) and the number of days from DOY_B_ to DOY_LE_, DOY_LMA_ and DOY_Chl_ (MP_LE_, MP_LMA_ and MP_Chl_, respectively). Panel (*c*) shows the determination of DOY_LE_, DOY_LMA_ and DOY_Chl_. A logistic regression curve is fitted to the seasonal variations in each parameter.

Since the 1980s, it has been recognized that leaf lifespan (LLS) is one of the most important characteristics for determining plant strategies related to carbon and nitrogen (N) use, and the coordinated relationships among LLS, leaf morphology and physiology have been extensively examined [1–4]. From a global survey, Wright *et al.* [5] showed that the ecophysiological variables form a spectrum of correlated traits, called the ‘leaf economics spectrum’ [5], which has recently been used in global vegetation modelling [6,7]. A theoretical study has explained the trade-offs between leaf traits from a cost-benefit perspective [8]. Leaves with a thick lamina generally have a low photosynthetic capacity and a long LLS because a long payback time for the high construction cost is required in terms of carbon economy (figure 1*a*).

Photosynthetic activity is lower in the winter, particularly at high latitudes [9] and high altitudes [10,11]. Therefore, in overwintering evergreen trees, the photosynthetically active period is shortened because of the cold winter [12,13], and these trees must enhance their desiccation tolerance via adaptations such as effective leaf osmotic adjustment during the winter season [14,15]. However, these trees have an advantage in that their photosynthesis can quickly restart immediately after the onset of the following spring [9]. The current-year leaves can make a substantial contribution to the total carbon gain in woody plants with a long LLS because the second-year leaves suffer from extensively reduced ribulose-1,5-bisphosphate carboxylase/oxgenage (Rubisco) activity [13] and self-shading owing to dense canopy [16]. However, the flushing of new leaves in overwintering evergreen trees generally occurs later than in winter-deciduous trees, probably to avoid damage by late spring frost [17–19]. Furthermore, in woody plants with a short LLS, including winter-deciduous trees, the photosynthetic capacity peaks around the time of full leaf expansion (LE) [20–23]. By contrast, evergreen trees generally have thick laminae and their photosynthetic capacity gradually develops over a month following the cessation of LE, resulting in a prolonged maturation period [21,24]. Therefore, the late leaf flushing and the prolonged leaf maturation period should shorten the photosynthetically active period in the current-year leaves of overwintering evergreen woody plants growing in warm-temperate forests, particularly near the latitudinal or altitudinal limit, where minimum temperature drops below zero in the mid-winter.

We examined how overwintering evergreen oaks in Japan compensate for the reduced carbon gain resulting from late leaf maturation and a shortened photosynthetic period in the current-year leaves. We addressed three possible hypotheses regarding the adaptive mechanism involved: hypothesis 1, hastening the timing of leaf-bud break and the full expansion of leaves; hypothesis 2, enhancing photosynthetic efficiency through leaf physiology; and hypothesis 3, increasing the total leaf area per shoot in shoot morphology. In this study, we report that the adaptive mechanisms in overwintering Japanese oaks mainly support hypothesis 2. We demonstrated that the universally accepted coordination among LLS, leaf physiology and morphology is partially cancelled out by specific constraints (late leaf maturation and overwintering). We compare the obtained data with global leaf trait relationships and show that the correlations between leaf variables in the regional or biome-level do not necessarily correspond with the global-scale analysis. Furthermore, we discuss the implications of the specific leaf characteristics of overwintering oaks in Japan.

## 2. Results and discussion

The study site is located almost at the northern limit of evergreen oaks in Japan. To understand the mechanisms in overwintering evergreen oaks, we selected adult trees of four evergreen species, *Quercus gilva* Blume, *Quercus glauca* Thunb., *Quercus acuta* Thunb. and *Quercus salicina* Blume, and as a comparison, of a winter-deciduous species, *Quercus serrata* Murray. We examined the leaf maturation process of the current-year leaves with respect to leaf size, leaf mass per area (LMA) and chlorophyll (Chl) accumulation for 1 year (figure 1*b*,*c*).

First, we evaluate hypothesis 1, related to leaf phenology. The calendar date of leaf-bud break (DOY_B_) varied by approximately two months among species (electronic supplementary material, figure S1). From the earliest DOY_B_ (1 April) to the latest (28 May), the order of bud break among the studied species was as follows: evergreen *Q. gilva*, deciduous *Q. serrata*, evergreen *Q. glauca*, evergreen *Q. acuta* and evergreen *Q. salicina*. Among the evergreen oaks, the DOY_B_ values were significantly negatively correlated with the LMA and leaf maturation periods (electronic supplementary material, table S1), indicating that early bud break contributes to developing a thicker and harder lamina. The evergreen *Q. gilva,* which had the earliest bud break, had the highest LMA and required 49 days and 96 days from leaf-bud break until the cessations of LE and LMA increase, respectively. In the other evergreens, the maturation periods required for LE and LMA were 19–33 days and 43–75 days, respectively (electronic supplementary material, table S2). As a result, the dates of completion for LE (DOY_LE_) and LMA increase (DOY_LMA_) in the evergreen *Q. gilva* were the second latest, indicating that the hastened leaf-bud break did not necessarily extend the photosynthetically active period in current-year leaves.

**Figure 2.**
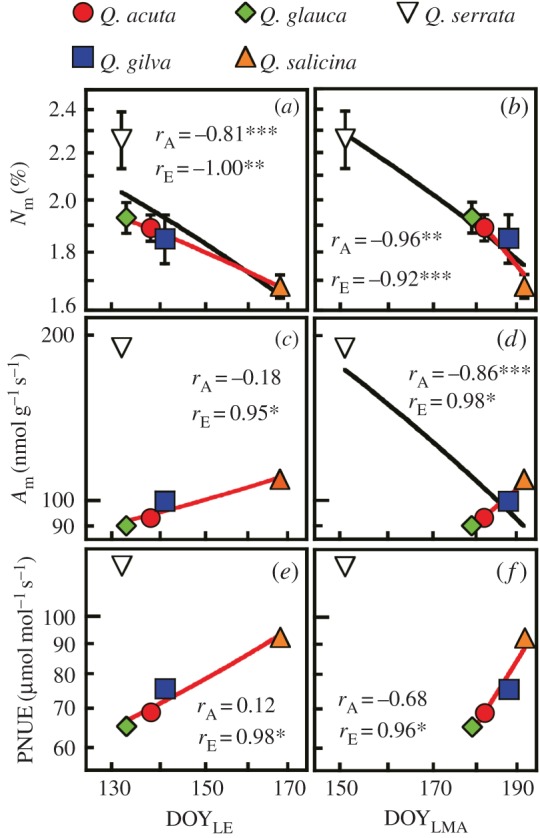
Relationships of leaf physiology with the dates of full leaf expansion (DOY_LE_) and of the developmental completion of the leaf mass per unit area (DOY_LMA_). (*a*,*b*) Mass-based nitrogen content (*N*_m_). (*c*,*d*) Mass-based photosynthetic capacity (*A*_m_). (*e*,*f*) Nitrogen-based photosynthetic capacity (PNUE). The data are shown for four evergreen (filled symbols) and a deciduous (open symbol) oak species. Evergreens with later leaf maturation had a lower leaf N content but a higher photosynthetic capacity. Note the log–log scale. The values of *r*_A_ and *r*_E_ represent Pearson's correlation coefficients among all of the species and among evergreen species, respectively (**p < 0.01, *p < 0.05, ***p < 0.15). The black and red lines represent regressions with *p* < 0.15 (as a marginally significant level) among all of the oak species and among the evergreen oak species, respectively. The bars represent ±1 s.d. (*n* = 7).

**Figure 3.**
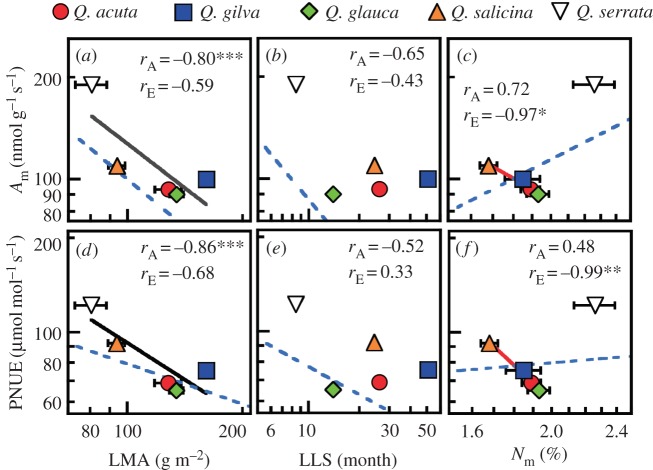
Relationships among the leaf mass per unit area (LMA), leaf lifespan (LLS), and leaf physiology. (*a*–*c*) Relationships between the mass-based photosynthetic capacity (*A*_m_) and the LMA, LLS and leaf mass-based nitrogen (*N*_m_), respectively. (*d*–*f*) Relationships between the nitrogen-based photosynthetic capacity (PNUE) and the LMA, LLS and leaf mass-based nitrogen (*N*_m_), respectively. The data are shown for four evergreen (filled symbols) and a deciduous (open symbol) oak species. Note the log–log scale. The values of *r*_A_ and *r*_E_ represent Pearson's correlation coefficients among all of the species and among evergreen species, respectively (**p < 0.01, *p < 0.05, ***p < 0.15). The black and red lines represent regressions with *p* < 0.15 (as a marginally significant level) among all of the oak species and among the evergreen oak species, respectively. The blue dashed lines represent regressions among the data in the GLOPNET dataset. The bars represent ±1 s.d. (*n* = 7).

Second, we evaluate hypothesis 2, related to leaf physiology. Among the four evergreen oaks, mass-based N (*N*_m_) was negatively correlated with DOY_LE_ and DOY_LMA_, whereas both mass-based assimilation rates (*A*_m_) and N-based assimilation rates (i.e. photosynthetic nitrogen use efficiency; PNUE) were positively correlated (figure 2). Consequently, evergreens with later leaf maturation dates had a lower leaf N content but higher mass-based and N-based photosynthetic abilities. The completion of LE and LMA increase in the winter-deciduous oak occurred on relatively early dates (before summer), whereas that in evergreens was later (in summer). LMA and LLS are key parameters for leaf economy [5]. However, LMA (figure 3*a*,*d*) and LLS (figure 3*b*,*e*) were not correlated with *A*_m_ and PNUE in evergreen oaks in Japan. *N*_m_ tended to be positively correlated with *A*_m_ and PNUE across all oaks (*r* *=* 0.72 and 0.48, respectively) (figure 3*c*,*f*), whereas *N*_m_ was negatively correlated with *A*_m_ and PNUE across the evergreens (*r* = −0.97 and −0.99, respectively). These results indicate that overwintering Japanese oaks have a particular use for N in their leaf physiology. Overall, our results support hypothesis 2, i.e. the evergreen oaks with later leaf maturation have a lower total leaf N content, but a higher fraction of this N is allocated to photosynthetic apparatus (i.e. high *A*_m_ and PNUE) than evergreen oaks with earlier leaf maturation, contributing to the enhanced carbon gain during the shortened growing season.

Third, we evaluate hypothesis 3, related to shoot morphology. An increased leaf area per shoot may overcome the shortage of carbon gain at the shoot level. However, in this study, the current-year leaf area per shoot and total leaf area per shoot were not correlated with leaf phenology (DOY_B_, DOY_LE_ and DOY_LMA_), photosynthetic capacity (*A*_m_ and PNUE), LLS or LMA (electronic supplementary material, table S1). Therefore, the shoot structure (hypothesis 3) is unlikely to contribute to the compensation for the shortened growing period in the current-year leaves.

**Figure 4.**
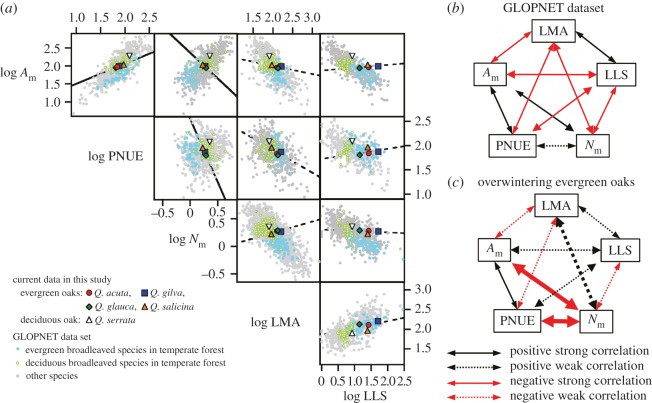
Relationships among leaf morphology, leaf physiology and leaf lifespan in overwintering oaks in this study and in the GLOPNET dataset. (*a*) Bivariate relationships (log_10_ transformed) between the light-saturated photosynthetic rates based on leaf dry mass (*A*_m_, nmol g^−1^ s^−1^) and based on leaf nitrogen (PNUE, μmol mol^−1^ s^−1^), the leaf nitrogen content based on leaf dry mass (*N*_m_, %), the leaf dry mass per area (LMA, g m^−2^), and the leaf lifespan (LLS, month) in the GLOPNET dataset. The light-blue filled circles and light-green open circles show data for evergreen and deciduous broad-leaves species in temperate forests in the GLOPNET dataset, respectively. The grey circles show other data in the dataset. Solid and dashed lines represent regressions with *p* < 0.05 and *p* ≥ 0.05 among evergreen oak species in this study, respectively. (*b*,*c*) Diagrams of correlations between the major parameters in the GLOPNET dataset (*b*) and overwintering evergreen oaks in Japan (*c*). The black and red arrows represent positive and negative correlations, respectively. The solid and dashed arrows represent strong and weak correlations, respectively. The correlations of *A*_m_, PNUE and LMA with *N*_m_ for the GLOPNET dataset are markedly different from those for the overwintering evergreen oaks in Japan, as shown by bold arrows in panel (*c*).

In order to evaluate whether the leaf variables in the overwintering oaks in Japan are included within the global-scale variations and whether the correlations between leaf variables correspond with the global-scale correlations, the leaf variables obtained in the evergreen oaks are compared with data from the worldwide database GLOPNET [5] (figure 4). The values of *A*_m_, PNUE, LMA and LLS were not significantly different between the evergreen oaks in Japan and evergreen-broadleaved trees in temperate forests worldwide (Tukey–Kramer HSD test; *A*_m_; *p* = 0.10, PNUE; *p* = 0.98, LMA; *p* = 0.83, LLS; *p* = 0.27). By contrast, the value of *N*_m_ in the evergreen oaks were significantly higher than those in evergreens in temperate forests (Tukey–Kramer HSD test; *p* = 0.002) and were not significantly different from deciduous broadleaved trees in temperate forests (Tukey–Kramer HSD test; *p* = 0.70), indicating that high *N*_m_ is a particular characteristic of the overwintering evergreen oaks in Japan. The high *N*_m_ will allow these trees to increase not only photosynthetic apparatus but also cell walls and anti-herbivore defence, contributing to their high LMA and long LLS. Across plant species worldwide, *N*_m_ is positively correlated with *A*_m_, and LLS and LMA are negatively correlated with *A*_m_ and *N*_m_. However, these correlations were not found among the evergreen oaks in Japan (figure 4; electronic supplementary material, table S1), indicating their particular strategy for N use within leaves. The globally positive correlation between *N*_m_ and *A*_m_ was entirely inverted in the overwintering evergreen oaks in Japan. Although *N*_m_ is often positively correlated with PNUE at the local scale [25], no clear correlation between PNUE and *N*_m_ was found at the global scale. As a possible reason for this pattern, our data suggest that N allocation within the lamina is variously adjusted by constraints within each biome. We hypothesize that the differences between the local and global scales are owing to differences in nitrogen allocation within leaves to overcome the constraints specific to each biome.

Our results indicate that the photosynthetically active period in the current-year leaves was limited not only by the late leaf flushing but also by the prolonged leaf maturation period, indicating a particular constraint for overwintering evergreens. To overcome this constraint in terms of carbon gain, Japanese oaks mainly adjust their leaf physiology (i.e. hypothesis 2), particularly with respect to N allocation within the lamina. Consequently, across Japanese overwintering oaks, the correlations among *N*_m_, *A*_m_ and PNUE exhibited the opposite relationships to the general global trends. Such an extreme phenomenon is rare; however, in a narrow biome, these correlations among leaf traits are occasionally weakened or otherwise altered from those in other biomes [26–29], indicating that there are occasionally biome-specific constraints. Such specific constraints are due to climate factors, such as temperature, drought and solar radiation [5], available nutrients in the soil [30,31] and biogeography [32]. Although worldwide meta-analysis is a useful tool, it should be noted that such biome-specific constraints can in some cases be hidden by averaging processes. N allocation within lamina will be controlled by these constraints, and as a result, the correlation between *N*_m_ and PNUE may be less visible in the GLOPNET dataset (figure 4). Local-scale relationships can differ from large-scale relationships. This study shows that constraints at the local scale result in varying trade-off correlations regarding carbon economy, even in physiological processes. Recently, a similar phenomenon was reported in a Mediterranean evergreen oak, *Quercus ilex* L. [33]. Therefore, thick lamina accompanied with delayed maturation may be a key factor for mediating local-specific leaf functional variations. Oaks have generally thick lamina, even in winter-deciduous trees [34,35]. More study for oak trees in the world is needed for understanding the correlations between worldwide leaf economics and local-specific leaf variations.

Among the overwintering evergreen oaks in Japan with a long LLS, the canopy leaves with a longer leaf maturation period had an earlier leaf-bud-break date, higher LMA, higher area-based N and chlorophyll content and higher area-based maximum assimilation rates than those with a shorter leaf maturation period, indicating that LMA was not correlated with LLS (electronic supplementary material, table S1). Therefore, early leaf-bud break contributed to enhance area-based carbon gain with a thick lamina (i.e. high LMA). In this study, LMA was more dependent on the carbon gain of single leaves than on LLS (electronic supplementary material, table S1). The number of examined trees in a species was limited in this study, because we examined physiological characteristics in the sun leaves of adult trees. However, the standard variations in this study were comparable with the published data in five adult trees of an evergreen oak (*Quercus myrsinifolia*) growing at this arboretum [13], indicating that our data will be representative in each tree species. Furthermore, note that the photosynthetic rates during the second year is conspicuously reduced by self-shading due to their dense canopy [16] and reduced Rubisco activity [13]. In the examined evergreen oaks, even when all canopy leaves are exposed to high light, the annual total carbon gain of the 1 year old shoots reached 19–59% of that of the current-year shoots (electronic supplementary material, table S3), because of the reduced photosynthetic capacities and defoliation (electronic supplementary material, figure S2). The fact indicates that a long LLS is not as contributing to the total carbon gain in individual leaves of evergreen oaks, because the self-shading severely reduces irradiance on the old-leaves surface. Therefore, the amounts of carbon gain in the current-year leaves before winter are critical for the total carbon gain in a single leaf and in a shoot even in the top-canopy shoots, because of the dense crowns. Substantial N was reserved within shaded (old) leaves and was trans-allocated to new leaves during leaf flushing [16]. LLS may thus be more dependent on N cycling within shoots than on LMA and the carbon gain in the shoots in overwintering Japanese oaks. Overall, our results show that the leaf variables have evolved strongly due to the affect of regional or biome-level constraints.

## 3. Material and methods

### 3.1. Plant materials

The study was conducted at an arboretum of the Forestry and Forest Products Research Institute (FFPRI) in Ibaraki Prefecture, Eastern Japan (36°00′ N, 140°08′ E, 20 m above sea level). The arboretum was established on a logged natural pine forest in 1978. Trees have never been fertilized in the arboretum. The annual mean precipitation is approximately 1300 mm, and the annual mean air temperature is approximately 14°C, with the highest temperature in August (approximately 35°C) and the lowest in January (approximately −7°C). From mid-December to early April in the winter, the mean daily air temperature is often below 5°C. We studied four evergreen oaks and a deciduous oak, which are common in the warm-temperate forests of Japan [36,37]. The study site is located near the northern limits of these evergreen oaks in Japan, whereas the range limit of the deciduous oak *Q. serrata* is farther north. We selected one or two planted individuals per species at sunny places along a path for the study. All trees were about 20 to 25 years old, and the heights and diameters at breast height were 10–15 m and 20–30 cm, respectively. All of the measurements were conducted on leaves from fully sun-exposed branches on the south side of the crown exposed to open light at approximately 4 m high. A rolling scaffold tower was used to access the sunlit canopies.

### 3.2. Leaf development observations

Leaf phenological observation began on 1 April 2004, and was continued every 1–3 days from the date of bud break until full LE. The schematic of the determination of leaf phenology is shown in figure 1*b,c*; DOY indicates the calendar date as counted from 1 January, and MP represents the day period from the day of leaf-bud break until the day of completion of leaf expansion, and LMA and Chl content increases. The day of leaf-bud break (DOY_B_) was determined when approximately 80% of the bud scales came off and the leaves began to emerge and unfold. After the bud break, we selected three sunlit branches for each species. The lengths of all of the new leaves in the branches were measured every one to two weeks until the cessation of LE (*n* = 16–43). In all of the examined oak species, almost all of the leaves in a shoot flushed simultaneously within a short period. To examine the increases in Chl and LMA during leaf development, we simultaneously collected seven current-year leaves from adjacent branches. The Chl contents per unit leaf area (*n* = 7) were measured using a chlorophyll meter (SPAD 502, Minolta, Tokyo, Japan) that was calibrated with the acetone extraction method for each species [38]. To determine the LMA (*n* = 7), leaf discs were cut with a borer from the collected lamina and the discs were dried at 70°C for 3 days and weighed. Following full LE, these measurements were continued every one to two months until the measured values were seasonally stable. In the timing of leaf physiological and morphological maturation, the three variables; leaf area, Chl and LMA were determined as follows. Logistic curves were fitted to seasonal variations in the leaf area, Chl and LMA. From the logistic curves, the completion dates of leaf maturation were determined when the leaf length reached 99% of the maximum and the Chl and LMA reached 95% of their maxima [24], called DOY_LE_, DOY_Chl_ and DOY_LMA_, respectively (figure 1*c*). In each parameter, the maturation periods were calculated as the day periods from DOY_B_ to DOY_LE_, DOY_Chl_ and DOY_LMA_, called MP_LE_, MP_Chl_ and MP_LMA_, respectively (figure 1*b*).

### 3.3. Physiological measurements following full leaf expansion

We selected five sunlit shoots. For the examined variables, the abbreviation and unit are shown in the electronic supplementary material, table S4 and the mean and 1 s.d. are shown in the electronic supplementary material, table S2. The seasonal variations in the leaf-area-based maximum photosynthetic rate (*A*_a_) were periodically measured between 9.00 and 14.00 on sunny days from May 2001 to April 2002 using an open-flow, portable measurement system (LI-6400, LI-COR, Lincoln, NE). In the summer seasons, the measurements of *A*_a_ were conducted between 9.00 and 11.00 to avoid the midday depression of photosynthesis. These measurements were conducted on a current-year leaf and a 1 year old leaf in each shoot. To measure the Chl content and LMA, we collected leaf discs from seven current-year leaves and seven 1 year old leaves of the adjacent shoots. After measurement of the LMA, the leaf nitrogen (N) content was measured using an NC analyser (NC-800, Sumigraph, Sumitomo-Kagaku, Osaka). The mass-based and the area-based N within the lamina (*N*_m_ and *N*_a_, respectively) and the mass-based and the N-based maximum photosynthetic rates (*A*_m_ and PNUE, respectively) were determined.

### 3.4. Leaf lifespan

The LLS was determined by a census from 2000 to 2002 of 10 sunlit shoots for each species. The fates of point-paint-marked leaves were followed every two months for 2 years. The LLS was calculated according to Ackerly & Bazzaz [39].

### 3.5. Shoot morphology

We examined shoot morphology to evaluate the potential carbon gain at the shoot level. The mean leaf area of individual leaves was randomly determined from more than 88 leaves collected from each species. The total leaf area in the current-year leaves (TLA_0_) and that in all of the leaves (TLA_all_) were determined based on the multiple of the numbers of leaves in each shoot (*n* = 10) and the mean leaf area. As an index of leaf allocation, the ratio of the total leaf area to the branch length in the current-year shoot (TLA_0.BL_) was examined.

### 3.6. Statistics and curve fitting

The significant variations among species studied were tested with ANOVA in each variable, and the significant differences in each species were compared with Tukey–Kramer HSD test (*p* < 0.05; electronic supplementary material, table S2). Pearson's correlation analysis was used to test the relationships between the pairs of traits (electronic supplementary material, table S1). The parameters were log_10_ transformed. As parameters for analysis, we used *A*_a_, *A*_m_, PNUE, Chl, *N*_a_, *N*_m_ and LMA when photosynthetic rates reached the seasonal peaks. Differences in *A*_m_, PNUE, *N*_m_, LMA and LLS between three groups, i.e. evergreen oaks in this study, evergreen and deciduous broadleaved species in temperate forest in the GLOPNET dataset, were tested by ANOVA followed by a Tukey–Kramer HSD test (*p* < 0.05). Statistical analysis and curve fitting were performed using the statistical software R (R Development Core Team 2010).

## Supplementary Material

Supplementary Information More detailed results, figures S1-S2, and tables S1-S4

## Data accessibility

To compare the leaf traits between Japanese evergreen oaks and worldwide trees, the GLOPNET dataset is archived in the electronic supplementary material in [5] (doi:10.1038/nature02403) and is available at www.nature.com/nature/journal/v428/n6985/suppinfo/nature02403.html.
