# Supplementary material for: Overwintering evergreen oaks reverse typical relationships between leaf traits in a species spectrum
Source: R Soc Open Sci. 2016 Jul 20;3(7):160276. doi: 10.1098/rsos.160276 (PMC4968473; doi:10.1098/rsos.160276)
Supplement: Supplementary Information More detailed results, figures S1-S2, and tables S1-S4 [file rsos160276supp1.docx]

**Supplementary Information**

**Title**: Overwintering evergreen oaks reverse typical relationships between leaf traits in a species spectrum

**More detailed results**

The developmental processes for leaf area, leaf mass per unit area (LMA), and chlorophyll (Chl) content per unit leaf area in the current-year leaves are shown in figure S1. How the leaf developmental processes were determined is shown in figure 1b, C. The abbreviations and units of variables related to the leaf traits are shown in Supplementary Table 3, and the mean values for each tree species are shown in Table S2. The timing of the leaf-bud break differed by approximately 2 months among species. Deciduous *Quercus serrata* showed a relatively early bud break in spring, and the leaf expansion was completed within 1 month. Evergreen *Q. gilva*, which had the earliest bud break, had the highest LMA and the longest maturation period for LMA, resulting in the second latest cessation of LMA increase among species. Therefore, early bud break was not necessarily related to early leaf maturation. These results result in the rejection of Hypothesis 1: i.e., in leaf phenology, hastening the timings of leaf bud break and the full expansion of leaves compensates for the delayed leaf maturation in the overwintering evergreen oaks in Japan.

The correlations between the measured variables were examined (Table S1). Across the evergreen oaks, a later bud break was significantly (*P* < 0.05) associated with a shorter maturation period for LMA, a lower area-based nitrogen (N) and Chl content, and a smaller LMA (i.e., a thin lamina). However, the day of bud break was not associated with the days of completion of leaf area or Chl and LMA development. A later cessation of leaf expansion was significantly (*P* < 0.05) associated with a low mass-based N (*N*_m_), a high mass-based photosynthetic rate (*A*_m_), and a high N-based photosynthetic rate (PNUE). This finding indicates that late leaf maturation results in a low accumulation of N within leaves*,* but high *A*_m_ and PNUE can compensate for low carbon gain during the growing season in the current-year leaves. As a result, the global positive correlation between *N*_m_ and *A*_m_ was reversed in overwintering evergreen oaks in Japan (figure 4). Although LMA and leaf lifespan (LLS) are well-known fundamental parameters of leaf economy, a negative correlation was found only between LMA and day of year of bud break (DOY_B_) (*P* < 0.05), indicating that late leaf-bud break is associated with a thin lamina. These results indicate that current-year leaves with more delayed leaf maturation have low leaf N concentrations but a high N allocation to photosynthetic enzymes within leaves, enhancing the carbon gain during the reduced growing period. These results support Hypothesis 2: i.e., in leaf physiology, the allocation of leaf N is adjusted to compensate for the reduced growing period.

In contrast, variables related to shoot morphology (TLA) were not significantly correlated with leaf physiology. Species with a late completion day for LMA (DOY_LMA_) or Chl (DOY_Chl_) did not have leafy shoots but, rather, leafless shoots (TLA) (*P* < 0.15), indicating that the shoot morphology did not strongly contribute to compensating for the shortened growing season in Japanese evergreen oaks. These results lead to the rejection of Hypothesis 3: i.e., shoot morphology is not adjusted to compensate for the reduced growing period in overwintering evergreen oaks in Japan.


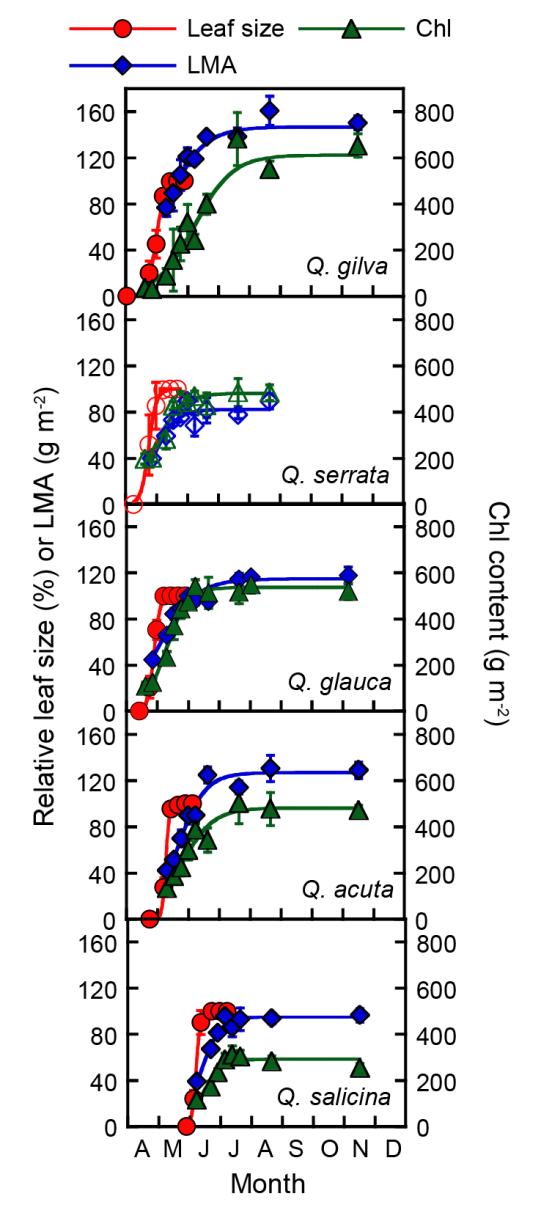


**Figure S1.** Seasonally development processes in the current-year leaves. Seasonal changes for relative leaf size (red circles), leaf mass per unit area (LMA) (blue diamonds), and chlorophyll (Chl) content (green triangles) in the current-year old leaves. *Q. serrata* is a winter-deciduous oak tree, and the other trees are overwintering evergreen oaks. The data are shown in order of bud break in the four evergreen oaks (closed symbols) and a deciduous oak (open symbols). Logistic curves were fitted. The bars represent ±1 standard deviation (leaf size, *n* = 14 to 43; LMA and Chl, *n* = 7). The evergreen *Q. gilva*, which had the earliest bud break, had the highest LMA but the longest maturation period for LMA, resulting in the second latest cessation of LMA increase among species.


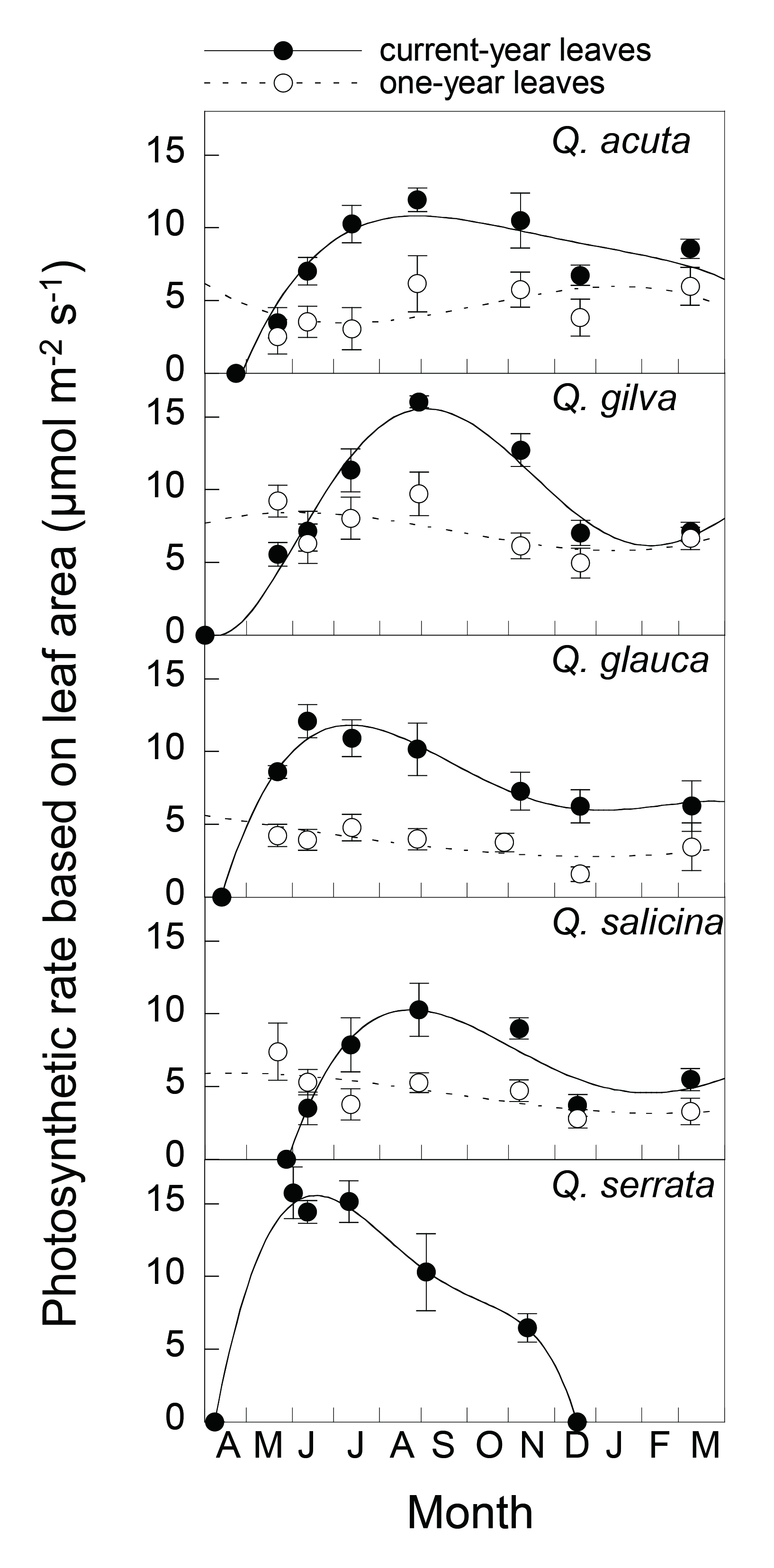


**Figure S2.** Seasonal changes for light-saturated photosynthetic rate based on leaf area in the current-year (closed symbols) and one-year leaves (open symbols). Fifth and fourth-order polynomial curves were fitted for the data in each current and one-year old leaves, respectively. We determined the values on the dates of bud break and of defoliation as 0. The bars represent ±1 standard deviation (*n* = 7).

| **Table S1.** Pearson's correlation for each pair of leaf traits. The data were log10 transformed. The coefficient coefficients (*r*) among all of the oaks (one deciduous and four evergreen oaks) are given in the upper-right section, and those among the four evergreen oaks are given in the lower-left section of the matrix. Significant or marginally significant coefficients of *P* < 0.05, *P* < 0.10, and *P* < 0.15 are shown in red, blue, and black bold letters, respectively. Non-significant coefficients (*P* ≥ 0.15) are shown in grey letters. The trait codes are shown in table S4. | | | | | | | | | | | | | | | | | | |
| --- | --- | --- | --- | --- | --- | --- | --- | --- | --- | --- | --- | --- | --- | --- | --- | --- | --- | --- |
|  | DOY_B_ | DOY_LE_ | DOY_Chl_ | DOY_LMA_ | MP_LE_ | MP_Chl_ | MP_LMA_ | *A*_a_ | *A*_m_ | PNUE | *N*_a_ | *N*_m_ | Chl | LMA | LLS | TLA_0_ | TLA_all_ | TLA_0.BL_ |
| DOY_B_ | DOY_B_ | **0.85** | 0.24 | 0.43 | **-0.91** | -0.68 | **-0.76** | **-0.89** | -0.16 | 0.08 | -0.72 | -0.65 | **-0.95** | -0.43 | 0.03 | -0.04 | 0.17 | 0.05 |
| DOY_LE_ | 0.84 | DOY_LE_ | 0.58 | 0.62 | -0.56 | -0.35 | -0.54 | -0.63 | -0.18 | 0.12 | -0.56 | **-0.81** | **-0.90** | -0.22 | 0.40 | -0.53 | -0.13 | -0.45 |
| DOY_Chl_ | 0.05 | 0.46 | DOY_Chl_ | **0.78** | 0.07 | 0.54 | 0.25 | -0.13 | -0.49 | -0.31 | 0.22 | **-0.75** | -0.20 | 0.48 | **0.95** | **-0.77** | 0.01 | **-0.88** |
| DOY_LMA_ | 0.49 | **0.88** | 0.77 | DOY_LMA_ | -0.21 | 0.20 | 0.24 | -0.55 | **-0.86** | -0.68 | 0.27 | **-0.96** | -0.29 | 0.60 | **0.82** | -0.38 | 0.45 | -0.62 |
| MP_LE_ | **-0.91** | -0.54 | 0.25 | -0.10 | MP_LE_ | **0.79** | **0.76** | **0.92** | 0.12 | -0.02 | 0.68 | 0.40 | **0.79** | 0.49 | 0.24 | -0.36 | -0.39 | -0.42 |
| MP_Chl_ | -0.81 | -0.49 | 0.53 | -0.04 | **0.87** | MP_Chl_ | **0.85** | 0.64 | -0.26 | -0.35 | **0.80** | 0.02 | 0.70 | 0.74 | 0.68 | -0.47 | -0.06 | -0.64 |
| MP_LMA_ | **-1.00** | -0.82 | 0.01 | -0.46 | **0.92** | 0.85 | MP_LMA_ | 0.51 | -0.50 | -0.65 | **0.99** | 0.03 | **0.85** | **0.91** | 0.51 | -0.10 | 0.27 | -0.38 |
| *A*_a_ | **-0.91** | -0.54 | 0.35 | -0.08 | **0.99** | **0.94** | **0.92** | *A*_a_ | 0.48 | 0.31 | 0.42 | 0.67 | 0.72 | 0.14 | -0.04 | -0.28 | -0.58 | -0.21 |
| *A*_m_ | 0.63 | **0.95** | 0.66 | **0.98** | -0.26 | -0.21 | -0.61 | -0.25 | *A*_m_ | **0.95** | -0.57 | 0.72 | -0.09 | **-0.80** | -0.65 | -0.04 | **-0.79** | 0.31 |
| PNUE | 0.71 | **0.98** | 0.59 | **0.96** | -0.37 | -0.32 | -0.69 | -0.36 | **0.99** | PNUE | -0.73 | 0.48 | -0.35 | **-0.86** | -0.52 | -0.23 | **-0.85** | 0.15 |
| *N*_a_ | **-0.99** | **-0.89** | -0.08 | -0.57 | **0.86** | 0.80 | **0.99** | **0.87** | -0.70 | -0.78 | *N*_a_ | 0.01 | **0.85** | **0.93** | 0.49 | 0.00 | 0.39 | -0.31 |
| *N*_m_ | -0.79 | **-1.00** | -0.51 | **-0.92** | 0.47 | 0.43 | 0.77 | 0.47 | **-0.97** | **-0.99** | 0.85 | *N*_m_ | 0.54 | -0.36 | -0.72 | 0.42 | -0.33 | 0.57 |
| Chl | **-0.97** | **-0.94** | -0.18 | -0.67 | 0.79 | 0.74 | **0.97** | 0.79 | -0.79 | **-0.85** | **0.99** | **0.91** | Chl | 0.59 | 0.04 | 0.21 | 0.14 | 0.04 |
| LMA | **-0.99** | -0.81 | 0.04 | -0.45 | **0.92** | **0.87** | **1.00** | **0.93** | -0.59 | -0.68 | **0.99** | 0.76 | **0.96** | LMA | 0.72 | -0.15 | 0.49 | -0.50 |
| LLS | -0.31 | 0.15 | **0.93** | 0.58 | 0.59 | 0.79 | 0.37 | 0.67 | 0.43 | 0.33 | 0.28 | -0.22 | 0.17 | 0.40 | LLS | -0.69 | 0.14 | **-0.89** |
| TLA_0_ | 0.01 | -0.52 | **-0.89** | **-0.86** | -0.40 | -0.46 | -0.05 | -0.43 | -0.76 | -0.68 | 0.07 | 0.59 | 0.20 | -0.07 | **-0.88** | TLA_0_ | 0.61 | **0.93** |
| TLA_all_ | -0.05 | -0.59 | -0.73 | **-0.88** | -0.36 | -0.28 | 0.03 | -0.35 | -0.80 | -0.73 | 0.16 | 0.65 | 0.29 | 0.02 | -0.72 | **0.96** | TLA_all_ | 0.31 |
| TLA_0.BL_ | 0.22 | -0.33 | **-0.87** | -0.74 | -0.58 | -0.62 | -0.26 | -0.61 | -0.61 | -0.51 | -0.14 | 0.41 | -0.01 | -0.28 | **-0.93** | **0.98** | **0.92** | TLA_0.BL_ |

| **Table S2.** Summary of the examined variables for each species (mean and 1 standard deviation). The trait codes and units are shown in table S4. The significant variations among species are tested with ANOVA in each variable, and the significant differences in each species are compared with Tukey-Kramer HSD test. Different letters accompanying data indicate significant differences among species (*P* < 0.05). | | | | | | | | | | | | | | | | |
| --- | --- | --- | --- | --- | --- | --- | --- | --- | --- | --- | --- | --- | --- | --- | --- | --- |
|  | Evergreen | | | | | | | | | | | |  | Deciduous | | |
| Trait | *Q. acuta* | | | *Q. gilva* | | | *Q. glauca* | | | *Q. salicina* | | |  | *Q. serrata* | | |
| *A*_a_ | 11.9 | ± | 0.8^a^ | 16.0 | ± | 0.4^b^ | 12.1 | ± | 1.1^a^ | 10.3 | ± | 1.8^a^ |  | 15.7 | ± | 1.8^b^ |
| *A*_m_ | 93.0 | ± | 6.3^a^ | 99.8 | ± | 2.5^a^ | 90.0 | ± | 8.4^a^ | 109.4 | ± | 19.4^a^ |  | 189.8 | ± | 13.8^b^ |
| PNUE | 68.9 | ± | 4.7^a^ | 75.5 | ± | 1.9^ab^ | 65.2 | ± | 6.1^a^ | 92.3 | ± | 16.2^b^ |  | 121.7 | ± | 13.7^c^ |
| *N*_m_ | 1.89 | ± | 0.05^a^ | 1.85 | ± | 0.09^a^ | 1.93 | ± | 0.06^a^ | 1.68 | ± | 0.04^b^ |  | 2.26 | ± | 0.13^c^ |
| *N*_a_ | 0.173 | ± | 0.011^a^ | 0.213 | ± | 0.017^b^ | 0.186 | ± | 0.011^a^ | 0.113 | ± | 0.007^c^ |  | 0.129 | ± | 0.006^c^ |
| LMA | 128.1 | ± | 10.2^a^ | 160.8 | ± | 5.9^b^ | 134.4 | ± | 5.5^a^ | 93.8 | ± | 4.7^c^ |  | 80.6 | ± | 7.7^d^ |
| Chl | 477.7 | ± | 72.0^ab^ | 550.2 | ± | 35.4^b^ | 513.1 | ± | 67.4^ab^ | 282.6 | ± | 25.1^c^ |  | 469.7 | ± | 21.0^a^ |
| LLS | 26.3 |  |  | 50.9 |  |  | 14.0 |  |  | 24.5 |  |  |  | 8.4 |  |  |
| DOY_B_ | 114 |  |  | 92 |  |  | 104 |  |  | 149 |  |  |  | 99 |  |  |
| DOY_LE_ | 138 |  |  | 141 |  |  | 133 |  |  | 168 |  |  |  | 132 |  |  |
| DOY_Chl_ | 192 |  |  | 209 |  |  | 162 |  |  | 197 |  |  |  | 159 |  |  |
| DOY_LMA_ | 182 |  |  | 188 |  |  | 179 |  |  | 192 |  |  |  | 151 |  |  |
| MP_LE_ | 24 |  |  | 49 |  |  | 29 |  |  | 19 |  |  |  | 33 |  |  |
| MP_Chl_ | 78 |  |  | 117 |  |  | 58 |  |  | 48 |  |  |  | 60 |  |  |
| MP_LMA_ | 68 |  |  | 96 |  |  | 75 |  |  | 43 |  |  |  | 52 |  |  |
| TLA_0_ | 0.0466 | ± | 0.0097^a^ | 0.0165 | ± | 0.0024^b^ | 0.0649 | ± | 0.0140^c^ | 0.0241 | ± | 0.0065^b^ |  | 0.0396 | ± | 0.0045^a^ |
| TLA_all_ | 0.0867 | ± | 0.0193^a^ | 0.0486 | ± | 0.0083^b^ | 0.0834 | ± | 0.0692^a^ | 0.0541 | ± | 0.0055^ab^ |  | 0.0396 | ± | 0.0045^b^ |
| TLA_0.BL_ | 0.189 | ± | 0.024^ab^ | 0.085 | ± | 0.020^c^ | 0.231 | ± | 0.012^a^ | 0.138 | ± | 0.064^b^ |  | 0.238 | ± | 0.058^a^ |

| **Table S3.** Estimated annual carbon gain of current-year and one-year old shoot (μmol m^-2^ year^-1^), calculated from light-saturated yearly seasonal changes of photosynthetic rate based on individual leaf area (figure S2) and total leaf area of current-year and one-year old shoot, respectively. | | | |
| --- | --- | --- | --- |
| Species | Current-year old shoot | One-year old shoot | One-year old/Current year old (%) |
| *Q. acuta* | 142.9 | 61.9 | 43% |
| *Q. gilva* | 47.7 | 28.2 | 59% |
| *Q. glauca* | 140.2 | 26.7 | 19% |
| *Q. salicina* | 62.6 | 27.0 | 43% |
| *Q. serrata* | 52.3 | 0.0 | 0% |

| **Table S4.** Abbreviations and units of the examined variables. | | |
| --- | --- | --- |
| Code | Definition | Unit |
| ***Leaf physiological parameters*** | | |
| *A*_a_ | Photosynthetic capacity based on leaf area | μmol m^-2^ s^-1^ |
| *A*_m_ | Photosynthetic capacity based on leaf dry mass | nmol g^-1^ s^-1^ |
| PNUE | Photosynthetic capacity based on leaf nitrogen (photosynthetic nitrogen use efficiency) | μmol mol^-1^ s^-1^ |
| *N*_m_ | Nitrogen content per unit leaf dry mass | % |
| *N*_a_ | Nitrogen content per unit leaf area | mol m^-2^ |
| LMA | Leaf dry mass per unit area | g m^-2^ |
| Chl | Chlorophyll a and b contents per unit area | μmol m^-2^ |
| LLS | Leaf lifespan | month |
| ***Leaf phenological and developmental parameters*** | | |
| DOY_B_ | Day of year in bud break | (date) |
| DOY_LE_ | Day of year in completion of leaf expansion | (date) |
| DOY_Chl_ | Day of year in completion of Chl accumulation | (date) |
| DOY_LMA_ | Day of year in completion of LMA development | (date) |
| MP_LE_ | Maturation day period from bud break to the completion of leaf expansion | day |
| MP_Chl_ | Maturation day period from bud break to the completion of Chl accumulation | day |
| MP_LMA_ | Maturation day period from bud break to the completion of LMA increase | day |
| ***Shoot morphological parameters*** | | |
| TLA_0_ | Total leaf area in a current-year shoot | m^2^ |
| TLA_ALL_ | Total leaf area in a shoot (all aged leaves) | m^2^ |
| TLA_0.BL_ | Total leaf area per branch length in a current-year shoot | m^2^ m^-1^ |
